# Editorial: Individual’s mechanics, movement and kinematics post-stroke

**DOI:** 10.3389/fbioe.2024.1430588

**Published:** 2024-06-04

**Authors:** Veronica Cimolin, Yih-Kuen Jan

**Affiliations:** ^1^ Department of Electronics, Information and Bioengineering, Politecnico di Milano, Milano, Italy; ^2^ IRCCS Istituto Auxologico Italiano, Piancavallo, Italy; ^3^ Rehabilitation Engineering Lab, Department of Health and Kinesiology, University of Illinois at Urbana-Champaign, Urbana, IL, United States

**Keywords:** stroke, rehabilitation, movement, kinematics, biomechanics

Disability after stroke is a major burden on society, due to its high incidence and prevalence. Among the priorities of rehabilitation programs, stroke rehabilitation aims to restore independence and improve patients’ quality of life. Dynamic balance, fall prevention and upper limb recovery are essential features for the clinical management of hemiparetic patients. In this context, the assessment of movement by means of quantitative movement analysis in hemiparetic post-stroke patients is key to planning rehabilitative interventions. Kinematic analysis facilitates the interpretation of the extent and mechanisms of motor recovery, and it has been increasingly applied in neurological research.

Although quantitative biomechanical approaches are objective, sensitive and quantitative, their associations with clinical measures have not been fully studied. Thus, the goal of the Research Topic was to provide a quantitative evaluation of the relationship between lower or upper extremity biomechanics and clinical scores to investigate in depth the motor dysfunction associated with stroke-related movement disabilities, which is critical to improving our understanding and expanding interventional strategies to minimize long-term consequences due to stroke.

We invited authors to submit their latest results in the field, in the form of original papers, reviews, or clinical cases, focusing mainly on biomechanics and movement analysis in stroke patients, rehabilitation programs for stroke patients and their quantitative outcomes and innovative data analysis and models to study the mechanisms of motor recovery; 9 papers were accepted for publication in this Research Topic and they are summarized in the following paragraphs.

The papers could be divided into two main categories: assessment of gait performance and upper limb during specific movements.

In terms of the assessment of gait performance, Li et al. studied the feasibility of muscle co-contraction using two EMG-based Co-Contraction Indices to approximate lower limb joint stiffness trends during gait in two individuals post-stroke patients. Abdollahi et al. conducted a systematic review of fall risk factors in the stroke community in order to identify their similarities and trends. Kantha et al. compared virtual reality (VR)-based skateboarding with walking at a comfortable walking speed on a treadmill in 20 young participants, in terms of kinematics and electromyographic activity of the trunk and legs; the authors demonstrated that the effect of VR skateboarding is particularly manifest when focusing on the supporting leg. Wang et al. investigated gait characteristics and fall risk in patients with cerebral small vessel disease (CSVD) and demonstrated that CSVD patients with seemingly normal gait and independent ambulation still have a high risk of falling. In particular, gait spatio-temporal kinematic parameters, gait symmetry, and gait variability were found to be important indicators for assessing high-fall risk. Sekiguchi et al. explored the differences in kinetic parameters of slow gait speed in patients with stroke across brain lesion sides. Lastly, with respect to the upper limb assessment category, Schwarz et al. examined inter-joint coordination in post-stroke patients during various upper limb movement tasks using parameters obtained from a wearable sensor, demonstrating that the kinematic parameters of the upper limb after stroke are largely influenced by the task. Cheng et al. investigated the kinematic components of the finger-to-nose test obtained from principal component analysis and the associations with upper extremity motor function in subacute stroke survivors. Li et al. provided an accurate interpretation and assessment of the underlying “motor control” deficits caused by stroke, using functional brain controllability analysis, based on electroencephalography and functional near-infrared spectroscopy, simultaneously recorded during a hand-clenching task. Goffredo et al. developed a predictive model for rehabilitation outcome at discharge assessed by the Motricity Index of the affected upper limb, based on multidirectional 2D robot-measured kinematics in individuals with subacute stroke.

It is evident from the articles in this Research Topic that improved performance in gait and upper limb motor skills leads to reduced risk of falls and better functioning in stroke patients. The articles in this Research Topic provide a foundation for the development of effective rehabilitation interventions to minimize the long-term consequences of stroke.

